# Association between Metabolic Syndrome, Smoking Status and Coronary Artery Calcification

**DOI:** 10.1371/journal.pone.0122430

**Published:** 2015-03-27

**Authors:** Yun-Ah Lee, Sung-Goo Kang, Sang-Wook Song, Jun-Seung Rho, Eun-Kyung Kim

**Affiliations:** 1 Department of Family Medicine, College of Medicine, The Catholic University of Korea, St. Vincent's Hospital, Suwon, Republic of Korea; 2 Health Promotion Center, College of Medicine, The Catholic University of Korea, St. Vincent's Hospital, Suwon, Republic of Korea; Children's National Medical Center, Washington, UNITED STATES

## Abstract

Coronary artery calcification (CAC), an indicator of coronary artery stenosis, is an independent risk factor of ischemic heart disease. Smoking increases the risk of metabolic syndrome (MS) and cardiovascular disease. Almost no previous studies have evaluated the combined effect of MS and smoking status on CAC. Therefore, in this study we examined the relationships between CAC, MS, and smoking. This study included 775 adult males without histories of cardiovascular disease who visited the Health Promotion Center at the University Hospital in Gyeonggi-do, Republic of Korea from January 2, 2010 to December 31, 2012. All subjects were screened for CAC by multi-detector computed tomography (MDCT). CAC increased significantly with age and body mass index (BMI). Among MS components, abdominal obesity and elevated fasting blood glucose were correlated with CAC. After adjusting for age and BMI, MS was associated with a 1.46-fold increase in CAC (95% CI:1.02-2.09), abdominal obesity was associated with a 1.45-fold increase (95% CI:1.04-2.04), elevated fasting blood glucose was associated with a 2-fold increase (95% CI:1.36-2.94), and MS and smoking combined were associated with 2.44-fold increase in CAC. Thus, the combination of smoking and MS had a greater impact on CAC than any single factor alone. MS is correlated with an increased risk of CAC, and a combination of MS and smoking is associated with even greater risk. These findings can be used to prevent cardiovascular disease in adults.

## Introduction

In recent years, the spread of a westernized lifestyle and diet have increased the prevalence of metabolic syndrome (MS) in Korea. MS is a complex disease involving abdominal obesity, impaired glucose tolerance, hypertension, and dyslipidemia that was first officially named in 1988, when it was called Syndrome X or Reaven’s Syndrome [1–5]. MS has also been referred to as insulin resistance syndrome and cardiovascular syndrome. Risk factors for MS include hyperglycemia, dyslipidemia, hypertension, and abdominal obesity. MS is diagnosed when at least three of five diagnostic criteria are satisfied [1,6,7]. MS increases the risk of coronary artery disease and mortality due to cardiovascular diseases and type 2 diabetes mellitus [8–13].

Risk factors of coronary artery disease include hypertension, increased LDL-cholesterol, diabetes mellitus, smoking, age, and family history of coronary artery disease. Although these risk factors are used to predict the occurrence of cardiovascular disease, their predictive value is as low as 60% [14–17]. Widely used methods for diagnosing coronary artery disease include Framingham score, myocardial scintigraphy, and echocardiography, although these methods have limitations. Thus, there are numerous ongoing studies focused on finding cost-efficient and effective methods with high sensitivity and specificity for predicting the occurrence of coronary artery disease [18,19].

The coronary artery calcification score (CACS) indicates the degree of calcification in coronary arteries. It is calculated using computer tomography (CT) to quantify the calcification score in each artery, and then adding all of those scores together. This calculation method was devised by Agatston et al. Higher CACS indicates higher risk of cardiovascular disease [20,21]. CACS is strongly correlated with the total amount of atherosclerotic plaque, being an independent risk factor of ischemic heart disease [22]. It is useful in the prevention and early detection of obstructive coronary artery disease [23]. Assessment of CACS can reduce the occurrence of ischemic heart disease because it becomes possible to diagnose and treat atherosclerosis in coronary arteries.

MS is closely related to smoking: smokers and former smokers are at increased risk of MS [24–26]. In addition, smoking increases low-density lipoprotein (LDL)-cholesterol and triglycerides (TG) in the blood and lowers high-density lipoprotein (HDL)-cholesterol, which results in increased risk of cardiovascular disease [27]. However, Cao et al. reported that smoking status was not associated with the prevalence of MS. They found that among MS components, elevated blood pressure, elevated fasting plasma glucose, elevated TG and overweight were significant predictors of CAC, but did not analyze risk of CAC according to smoking status [28]. Previous studies have documented associations between smoking and CAC. The accumulation of CAC is accelerated by smoking and decreases after smoking cessation [29]. Previous trials indicated that Framingham risk factors, especially age, LDL-cholesterol, blood pressure and present smoking, are predictors of CAC in middle-aged to elderly men and women in the general population without prevalent CAC at baseline [30]. The risk of CAC increases with increasing numbers of MS components [28]. Bonora et al. reported that age, smoking, HbA1c and the presence of MS were independent risk factors for cardiovascular disease (CVD) [31].

Very few studies have explored the combined effect of MS and smoking on CAC. The purpose of the present study was to determine whether there are associations between CACS, measured by multi-detector computer tomography (MDCT), and each risk factor of MS. We also explore the potential combined effect of MS and smoking on CAC.

## Methods

### Study Population

The initial study population included 887 adult males who visited the Health Promotion Center of the University Hospital in Gyeonggi-do, Republic of Korea from January 2, 2010 to December 31, 2012 for check-ups and coronary artery MDCT. Of these 887 subjects, 112 were excluded due to cardiovascular diseases such as angina pectoris and myocardial infarction, resulting in a final study population of 775 individuals.

### Ethics Statement

This study was implemented in accordance with ethical and safety guidelines upon the approval of the Institutional Review Board of The Catholic University of Korea, St. Vincent’s Hospital (IRB approval number: VCRASI0112). The study was exempted from requirements for written informed consent because we reviewed the health screening data and medical record retrospectively. All data records were de-identified and analyzed anonymously. The IRB approved this consent procedure.

### Basic Survey and Physical Examination

Before undergoing physical examinations, subjects self-reported their medical histories including hypertension, diabetes mellitus, angina pectoris, and myocardial infarction. They also provided information about medication history, as well as smoking status, alcohol intake, and exercise habits. Body mass index (BMI) and body composition were measured with the InBody 3.0 device (Biospace, Korea), which uses bioelectrical impedance analysis (BIA). In accordance with World Health Organization (WHO) guidelines, waist circumference was measured between the lower border of the rib cage and the top of the iliac crest as the subject exhaled, using a tape measure and rounding to the nearest tenth of a centimeter. Blood pressure was measured with an automatic manometer after each subject was allowed to relax for at least 10 minutes.

### Biochemical Analysis

Blood samples were collected on the day of the examination after at least 8 hours of fasting. Flow cytometry with an automatic clinical chemistry analyzer (Sysmex XE-2100; Japan, and Hitachi 7600; Japan) was used to analyze fasting blood glucose, total cholesterol, TG, HDL-cholesterol and LDL-cholesterol levels for each sample.

### Coronary Artery Calcification Test

A 64-Slice MDCT (Sensation 64, Siemens, Erlangen, Germany) was used for the CACS. All subjects fasted at least 6 hours prior to the examination and those with heart rates faster than 65 beats-per-min took β-Blocker to lower their heart rates before the test. Subjects were injected with 60–70 ml of nonionic contrast (Ultavist 370, Schering, Germany) followed by 40 ml of saline. After obtaining images with electrocardiogram synchronization, CAC was quantified using a reconstruction program (Wizard, Siemens, Erlangen, Germany). CACS was calculated according to the standards of the Agatston Score. This method divides the coronary artery into four segments (right, left main, left anterior, and left circumflex), quantifies each artery, and adds all four scores to calculate the CACS. For the present study, CAC was considered present if the CACS was greater than zero.

### Diagnostic Criteria for Metabolic Syndrome

Metabolic syndrome (MS) is present when three or more of the following criteria are satisfied, as suggested by the American Heart Association (AHA) and the National Heart, Lung, Blood Institute (NHLBI):

1. Abdominal obesity: Waist circumference ≥90cm (men) and ≥80cm (women) for Asians
2. Triglycerides ≥150 mg/dL or medicated to treat this condition
3. HDL-cholesterol <40mg/dL (men), <50mg/dL (women) or medicated to treat this condition
4. Fasting blood glucose ≥100mg/dL or medicated to treat this condition
5. Blood pressure ≥130/85mmHg or medicated to treat this condition

### Statistical Analysis

Data were analyzed with SPSS version 18.0 for Windows. We calculated frequency, percentage, average and standard deviation of subject characteristics and MS components. The χ2-test was used to analyze differences in CAC dispersion depending on subject characteristics, the effect of MS, and/or smoking status. Factors related to CAC were analyzed using CAC presence as the dependent variable. To detect an odds ratio of 2.0 using a two-tailed test with a significance level of 5 percent and a power of 95 percent, we needed 689 individuals [32]. Odds ratios and 95% confidence intervals were calculated through multivariate logistic regression analysis. P-values less than 0.05 were considered statistically significant.

## Results

### Baseline Characteristics of Subjects

A total of 381 (49.2%) subjects were obese based on BMI. The majority of the subjects were former smokers (330; 42.6%), drank alcohol at least once a week (579; 74.7%), and regularly exercised at least once a week (297; 38.3%). The mean age of subjects in the present study was 52.5 years, and mean CACS was 35.0±155.6. There were 266 subjects (34.3%) with CAC (CACS>0). CAC increases with age (P<0.001). Subjects younger than 40 years old had a calcification rate of 3.8%, while subjects in their 40s, 50s, and 60s had rates of 17.3%, 41.9% and 54.8%, respectively. Of the individuals categorized as “normal weight” according to BMI, only 46 (26.4%) exhibited CAC, while 150 obese subjects (39.4%) exhibited calcification. These differences were statistically significant (p = 0.008). There were no associations between CAC and alcohol intake, exercise, or smoking habits (Table 1).

**Table 1:** Baseline Characteristics of study populations.

Variables	CACS(0)	CACS(>0)	*p*
	N(%)	N(%)	
**Age(years), n(%)**			**<0.001**
** <40**	**51(96.2)**	**2(3.8)**	
** 40–49**	**206(82.7)**	**43(17.3)**	
** 50–59**	**172(58.1)**	**124(41.9)**	
** 60≤**	**80(45.2)**	**97(54.8)**	
**BMI (kg/m^2^)**			**0.008**
** Normal(18.5–22.9)**	**128(73.6)**	**46(26.4)**	
** Overweight(23.0–24.9)**	**150(68.2)**	**70(31.8)**	
** Obesity(25.0≤)**	**231(60.6)**	**150(39.4)**	
**Alcohol drinking, n(%)**			**0.192**
** ≥1 times/wk**	**388(67.0)**	**191(33.0)**	
** ≤1times/monthor N Alcohol drinking**	**121(61.7)**	**75(38.3)**	
**Exercise (%)**			**0.223**
** Regular ≥1times/wk**	**184(62.0)**	**113(38.0)**	
** Irregular**	**209(68.3)**	**97(31.7)**	
** No exercise**	**116(67.4)**	**56(32.6)**	
**Smoking status**			**0.202**
** Current smoker**	**215(67.6)**	**103(32.4)**	
** Former smoker**	**206(62.4)**	**124(37.6)**	
** Non smoker**	**88(69.3)**	**39(30.7)**	

BMI: body mass index

### Association between MS and CACS

There was a higher rate of CACS in individuals with MS (128; 44.0%) than in those without (138; 28.5%) (p<0.001). In addition, CACS was more prevalent in subjects who exhibited abdominal obesity (129; 40.4%) compared to those who did not (137; 30.0%) (p = 0.002). A higher rate of CACS was also observed in subjects with high blood pressure (182; 39.9%) compared to those with normal blood pressure (84; 26.3%) (p<0.001). Finally, a higher rate of CACS was observed in subjects with elevated fasting blood glucose (84; 52.5%) compared to those who had normal fasting blood glucose (182; 29.6%) (p<0.001) (Table 2).

**Table 2:** Association between Metabolic Syndrome Components and CACS.

Variables		CACS(0)	CACS(>0)	*p*
		N(%)	N(%)	
**MS**	**No**	**346(71.5)**	**138(28.5)**	**<0.001**
	**Yes**	**163(56.0)**	**128(44.0)**	
**WC≥90 cm(male)**	**No**	**319(70.0)**	**137(30.0)**	**0.002**
	**Yes**	**190(59.6)**	**129(40.4)**	
**BP ≥130 /85(mmHg)**	**No**	**235(73.7)**	**84(26.3)**	**<0.001**
	**Yes**	**274(60.1)**	**182(39.9)**	
**FBS ≥100 mg/dL**	**No**	**433(70.4)**	**182(29.6)**	**<0.001**
	**Yes**	**76(47.5)**	**84(52.5)**	
**HDL-C <40mg/dL (male)**	**No**	**299(64.7)**	**163(35.3)**	**0.537**
	**Yes**	**210(67.1)**	**103(32.9)**	
**TG ≥150 mg/dL**	**No**	**307(67.8)**	**146(32.2)**	**0.167**
	**Yes**	**202(62.7)**	**120(37.3)**	

WC: waist circumference, BP: blood pressure, FBS: fasting blood sugar, HDL: high density lipoprotein, TG: triglyceride, CACS: coronary artery calcification score

### Relationship between MS Components and CAC

A multivariate logistic regression analysis was performed to determine the odds ratios of CAC according to MS components. Even after adjusting for age and BMI (Model 2), abdominal obesity was associated with a 1.45-fold increase in the odds ratio of CAC (95% CI:1.04–2.04). High fasting blood glucose was a statistically significant risk factor of CAC, as it resulted in a two-fold increase in the odds ratio of CAC (95% CI:1.36–2.94) (Table 3).

**Table 3:** Relationship between Metabolic Syndrome Components and Coronary Artery Calcification.

	Model 1 OR(95%CI)	Model 2 OR(95%CI)
**WC≥90 cm(male)**	**1.58(1.17–2.13)**	**1.45(1.04–2.04)**
**BP ≥130 /85(mmHg)**	**1.86(1.36–2.54)**	**1.17(0.83–1.67)**
**FBS ≥100 mg/dL**	**2.63(1.84–3.75)**	**2.00(1.36–2.94)**
**HDL-C <40mg/dL (male)**	**0.90(0.66–1.22)**	**0.73(0.52–1.02)**
**TG ≥150 mg/dL**	**1.25(0.93–1.69)**	**1.17(0.89–1.56)**

Model 2 is adjusted with age, BMI by multivariable analysis

WC: waist circumference, BP: blood pressure, FBS: fasting blood sugar

HDL-C: high density lipoprotein Cholesterol, TG: triglyceride

OR:odds ratio, CI:confidence interval

### Association between MS, Smoking Status and CAC

Subjects were divided into four groups based on presence of MS and smoking status. Of the non-smokers without MS, 24 (25.8%) had CAC. In addition, 114 (29.2%) of the smokers without MS, 15 (44.1%) of the non-smokers with MS, and 113 (44.0%) of the smokers with MS had CAC. The differences between these four groups were statistically significant (p<0.001) (Table 4).

**Table 4:** Association between Metabolic Syndrome and/or Smoking Status and Coronary Artery Calcification.

Variables	CACS(0)	CACS(>0)	*p*
	N (%)	N (%)	
**Group**			**<0.001**
** MS(-)Smoking(-)**	**69(74.2)**	**24(25.8)**	
** MS(-)Smoking(+)**	**277(70.8)**	**114(29.2)**	
** MS(+)Smoking(-)**	**19(55.9)**	**15(44.1)**	
** MS(+)Smoking(+)**	**144(56.0)**	**113(44.0)**	

MS(-):no metabolic syndrome, MS(+): metabolic syndrome

Smoking(-): non-smoker, Smoking(+): current smoker and former-smoker

### Risk of CAC according to MS and Smoking Status

**Fig 1:**
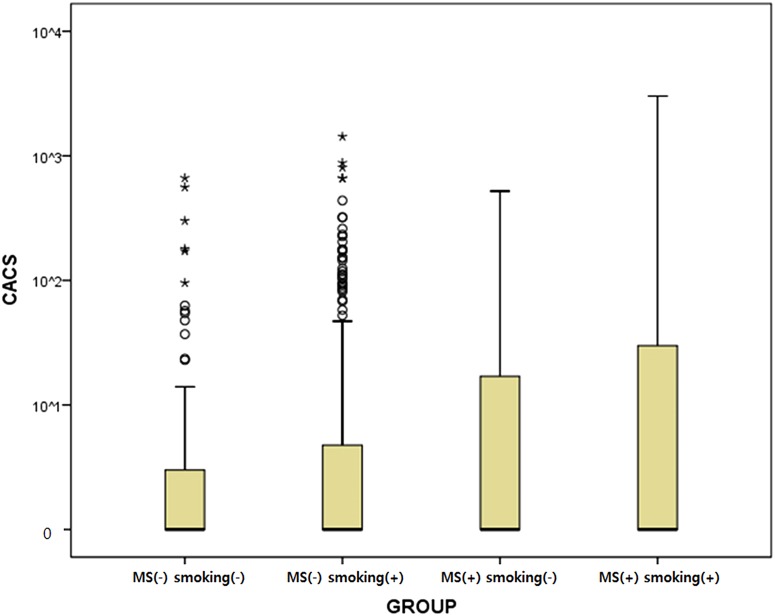
The distribution of Coronary Artery Calcification Score (CACS) according to Metabolic Syndrome (MS) and Smoking Status.

A logistic regression analysis was used to explore relationships between CAC and MS and between CAC and smoking. MS was associated with CAC (OR: 1.46; 95% CI: 1.02–2.09), after adjusting for age and BMI, while smoking was not (OR: 0.90; 95% CI: 0.75–1.08) (Table 5). However, smoking combined with MS was significantly associated with CAC (OR 2.44; 95% CI:1.39–4.21) (Table 6). Interaction terms for smoking-MS were not significant (p = 0.6997) (Fig. 1).

**Table 5:** Odds Ratios of Metabolic Syndrome and Smoking to Coronary Artery Calcification.

	Model 1 OR(95%CI)	Model 2 OR(95%CI)
**MS**	**1.95(1.44–2.65)**	**1.46(1.02–2.09)**
**Smoking**	**1.12(0.74–1.69)**	**0.90(0.75–1.08)**

Model 2 is adjusted with age, BMI by multivariable analysis

MS:metabolic syndrome, OR:odds ratio, CI:confidence interval

**Table 6:** Odds ratios for Coronary Artery Calcification according to Metabolic Syndrome and Smoking Status.

	Model 1 OR(95%CI)	Model 2 OR(95%CI)
**MS(-) smoking(-)**	**reference**	**reference**
**MS(-) smoking(+)**	**1.18(0.71–1.98)**	**1.36(0.79–2.34)**
**MS(+) smoking(-)**	**2.27(0.99–5.16)**	**2.15(0.90–5.14)**
**MS(+) smoking(+)**	**2.26(1.33–3.82)**	**2.44(1.39–4.21)**

Model 2 is adjusted with age, BMI by multivariable analysis

MS: metabolic syndrome, OR: odds ratio, CI: confidence interval

## Discussion

In this study, we investigated the relationships between CAC, MS, and smoking in adult Korean males with no histories of disease. CAC increased with age, particularly in subjects over 50, being in agreement with those findings reported by Choi et al. in 1999 [23]. After adjusting for age and BMI, MS significantly increased the odds ratio of CAC, as did abdominal obesity and elevated fasting blood glucose.

CAC is an indicator of atherosclerosis. It is an independent risk factor of ischemic heart disease [22,33]. The more severe the calcification, the more likely that coronary arterial disease will occur [34]. A 3.5-year follow-up study of 5635 asymptomatic patients reported that patients without CAC (CACS: 0) had very low rates of cardiovascular disease, while patients with high levels of CACS were four times more likely to have coronary artery disease [35]. A 3-year follow up study focused on coronary artery disease in 2000 asymptomatic adult subjects found that when CAC is present, the onset rate of coronary artery disease increases 10.5 times in men and 2.6 times in women [36]. A Korean study also reported that CACS was effective at prevention and early detection of obstructive coronary artery disease [23]. Moreover, smoking increased the risk for cardiovascular disease by increasing LDL-cholesterol and TG and lowering HDL-cholesterol levels in blood [27].

Wong et al. reported that subjects with MS had a higher CACS and CAC deposit rate than subjects without MS [20]. A study by Kim et al., conducted in a sample of adults with no medical history of obstructive coronary artery disease and with none of the typical symptoms of coronary artery disease, reported that MS was a risk factor of CAC [37]. These findings suggest that patients with MS have higher incidence of cardiovascular disease. Of MS components, abdominal obesity and high fasting blood glucose are significant risk factors of CAC (CACS>0). Abdominal obesity is strongly correlated with cardiovascular disease [38]. Rather than viewing general obesity as an independent factor of cardiovascular disease, it is more meaningful to consider abdominal obesity as a part of MS [39]. Thus, lifestyle changes are important for preventing cardiovascular disease in individuals with increased waist circumference, regardless of whether or not they are obese according to BMI. MS component, especially elevated TG and decreased HDL were not correlated with CAC in our study. However, in a previous trial by Cao et al., elevated TG was a significant predictor of CAC [28]. This discrepancy might be related to ethnic or dietary differences.

In this study, we found no significant correlation between smoking and CAC. This may be due to the fact that we failed to consider each subject’s pack-years, and instead used current smoking status. We also did not take into account alcohol intake, self-reporting bias, or stress associated with smoking. Thus, a correlation between smoking and CAC may become apparent if other factors are considered.

However, a combination of MS and smoking did influence on the odds ratio for CAC. Smokers without MS had an odds ratio of 1.36 for CAC, while non-smokers with MS had an odds ratio of 2.15, and smokers with MS had a significantly higher odds ratio of 2.44. Thus, the combination of MS and smoking results in a greater increase in the odds ratio of CAC compared to MS or smoking alone. Our results also suggest that smoking combined with MS increases the onset rate of CAC, when compared to smoking without MS. Although several previous studies have evaluated the associations between MS and CAC and smoking and CAC, almost no studies have explored the combined effect of MS and smoking status on the risk of CAC. MS and smoking are both independent risk factors for cardiovascular disease [31]. However, the available data regarding the combined effects of MS and smoking are limited. Several previous studies showed associations between smoking, MS and carotid arteriosclerosis. Ishizaka et al. found that both former and current smokers had an increased prevalence of MS. MS was found to be a risk factor for carotid plaque. Cigarette smoking increased the prevalence of carotid plaque in men without MS after adjusting for other cardiovascular risk factors [25].

MS predicts cardiovascular disease and mortality [1,8]. Wong et al. demonstrated that individuals with MS have higher likelihood and prevalence of coronary atherosclerosis, as measured by CAC, than persons without MS [40,41]. Furthermore, smoking increases endothelial dysfunction and atherosclerotic plaque formation by reducing endothelial nitrous oxide (NO) production, elevating serum levels of LDL-cholesterol. Smoking also promotes platelet aggregation and coagulation activity, increasing the overall risk of thrombus formation [42]. Therefore, the combination of MS and smoking can increase cardiovascular risk more than MS or smoking alone.

There are several possible explanations for the association between MS, smoking and CAC. Smoking promotes vascular changes, leading to insulin resistance by decreasing glucose uptake by skeletal muscle [43]. It is possible that smoking induces an increase in proinflammatory cytokines that enhance leukocyte-endothelial cell interaction, leukocyte recruitment and activation of proatherogenic substances and oxidative modification of lipids [44]. Additional studies are needed to investigate the mechanism in which smoking increases CAC in persons with MS.

Our study has several limitations. First, our results may not be applicable to the general public, as the subjects were all adult males who visited the health promotion center at a university hospital. There may be selection bias because subjects voluntarily visited the center and therefore may care more about their health than members of the general public. Second, we did not include data such as pack-years, or detailed information about alcohol intake and exercise habits.

In summary, MS is correlated with increased risk of CAC and that a combination of MS and smoking was associated with an even greater risk of CAC. These findings may be useful to prevent cardiovascular disease in adults.
